# The association between implant design, age, sex and the rate of major reoperation in patients undergoing primary total hip replacement: A retrospective study of UK National Joint Registry and Hospital Episodes Statistics data

**DOI:** 10.1371/journal.pmed.1004538

**Published:** 2025-11-26

**Authors:** Josh N. Lamb, Adrian Sayers, Jeremy Mark Wilkinson, Hemant Pandit, Michael R. Whitehouse

**Affiliations:** 1 Centre for Hip Surgery, Wrightington Hospital, Wigan, United Kingdom; 2 Musculoskeletal Research Unit, Translational Health Sciences, Bristol Medical School, Southmead Hospital, Bristol, United Kingdom; 3 Division of Clinical Medicine, School of Medicine and Population Health, University of Sheffield, Sheffield, United Kingdom; 4 Leeds Institute of Rheumatic and Musculoskeletal Medicine (LIRMM), University of Leeds, C/O Chapel Allerton Hospital, Leeds, United Kingdom; 5 National Institute for Health Research Bristol Biomedical Research Centre, University Hospitals Bristol and Weston NHS Foundation Trust and University of Bristol, Bristol, United Kingdom; University of Adelaide School of Medical Sciences: The University of Adelaide Adelaide Medical School, AUSTRALIA

## Abstract

**Background:**

Implant revision is an operation with exchange of implants, and is used as a standard outcome after total hip replacement (THR), but may not fully represent the patient experience after a THR. Major reoperation (hereafter referred to as ‘reoperation’) without revision of implants can also lead to increased patient morbidity and mortality, and most commonly occurs when the femur fractures around an implant (postoperative periprosthetic femoral fractures; POPFF) and is treated with fixation and the implant is left in place. Reliance on revision metrics that do not capture these reoperations has led to large-scale underreporting of reoperations in THR, and is likely to have affected implant performance estimates, which have guided national policy and implant selection. It is important to include these additional reoperations when estimating treatment success to guide innovation and clinical practice. We aimed to estimate the incidence of reoperation following primary THR.

**Methods and findings:**

We performed a large national cohort study on a mandatory, prospective database, the National Joint Registry, linked to Hospital Episode Statistics. All linkable primary THRs using recently available implants, with highest safety ratings between 01/01/2010 and 31/12/2020, were included. Major reoperation was defined as the first revision for any cause or fixation of POPFF and was identified using a combination of procedural and diagnosis codes. We identified 372,967 THRs representing 2,127,464 prostheses years at risk with a median follow-up time of 5.39 years (range 0 to 12.1 years). A total of 8,043 reoperations were identified that had been surgically treated by revision for any cause or fixation of POPFF. The incidence of reoperation was 3.78% (95% confidence interval [CI 3.70%, 3.86%]) per 1,000 prostheses years in comparison to 3.00% (95% CI [2.93%, 3.07%]) per 1,000 prostheses years when using conventional revision only outcomes. Cumulative incidence of major reoperation at 10 years was 3.1% (95% CI [3.0%, 3.1%]). Cumulative reoperation estimates were stratified by age and sex. In men aged 68 years and older, collared cementless stems performed better than cemented stems and in women aged 75 years and older, the relationship was reversed. Residual differences in patient characteristics may affect the accuracy of the estimates.

**Conclusions:**

Treatment failure after THR has been underrepresented by revision-only estimates. Major reoperation rates in older men were lowest with cementless collared stems, and in older women, reoperation rates were lowest with cemented polished taper stems made of stainless steel. These results prompt a review of the current implant guidance for hip replacements in older patients.

**Level of evidence:**

III (Retrospective cohort study).

## Author summary

**Why was the study done?:**

- More than 1 million hip replacements a year are performed globally, so even small differences in implant performance can lead to a large number of patients needing additional surgery.
- Current guidelines for implant selection don’t consider all adverse events experienced by patients that lead to a return to theatre for a major reoperation.
- This study aimed to provide a more representative understanding of the incidence of major reoperation for patients of different ages and sexes with different implants to help guide treatment decisions.

**What did the researchers do and find?:**

- Hip replacements performed in England were matched to health data on hospital admissions to find patients who were treated with either revision or fixation of the fracture without exchange of implants. Only cases from the last decade using implants which were commonly used and had highest safety rating were included to make the data more representative of current practice and decision-making.
- The most common reason for reoperation overall was periprosthetic fracture of the femur (POPFF). In all patients older than 75 years, POPFF accounted for almost half of the indications for reoperation.
- This study found that overall, cementless collared implants performed as well as the most commonly used cemented femoral implants. Men of 68 years of age and above with cementless collared stems incurred fewer major reoperations than those with the best-performing cemented stem. Females with a cemented femoral stem over 75 years old incurred fewer major reoperations than those with collared cementless stems. This suggests the need for implant selection based on patient sex rather than uniform guidelines based only on patient age.

**What do these findings mean?:**

- These results suggest that there is an interaction between the design of femoral implant, patient age, and sex. Current age-based guidance on implant use may lead to excess reoperations in older men undergoing primary total hip replacement (THR).
- Health care providers and researchers should re-evaluate implant selection to optimise patient outcomes and reduce the risk of major reoperation following THR.
- Accuracy of findings may be limited by differences in patient characteristics between the compared groups.

## Abstract 3

Josh N Lamb and colleagues estimate the cumulative incidence of first-time reoperation following primary total hip replacement, including revision for any cause or fixation of the femur for postoperative periprosthetic femoral fracture. They considered different age groups, sex, and implant types to help guide treatment decisions.

## Introduction

(THR) is a common and effective treatment for severe hip arthritis and most hip replacements can be expected to last longer than 20 years [1]. More than 1 million THRs are performed across the world each year [2], and small improvements in outcomes can have a very large effect on patient health and resource use in healthcare systems [3]. Hip replacements can fail for a range of reasons and recent evidence suggests that the most common cause of major reoperation is postoperative periprosthetic fracture of the femur (POPFF) [4]. The assessment of the success of THR is generally based on a reoperation where the implants are added, modified or exchanged, which is often described as a revision; however, methods using revision as an outcome may miss up to a fifth of major reoperations if surgeons choose to keep the original implants in place when performing reoperation [4,5]. Fixation is a common and successful approach for treatment of POPFF [6], but nevertheless, patients may still experience equivalent risk of complications and death to those undergoing revision [7]. Therefore, a patient-centred evaluation of THR success should include all major reoperations regardless of whether the surgeon chooses to revise the implants or not as part of that procedure.

Hip replacements come in many forms and the traditional grouping is based on whether the implant is fixed to bone with or without cement [2]. This principle has dominated the discourse of hip surgeons and has been combined with revision-only evaluation of implant performance [8] to form the foundation of national guidance stipulating the use of any cemented femoral implant in hip replacements for patients over the age of 70 years in England [9]. This principle is based, at least in part, upon the observation that periprosthetic osteolysis due to release of wear particle debris is the main reason for failure, and for which the main treatment is revision surgery [10]. However, as the population ages and prosthesis design and materials have evolved, other reasons for repeat surgery have become more prominent and this thinking may no longer be valid [4,11–13]. Further, implant design rather than the presence or absence of cement are likely to influence the risk of POPFF [14], which is the leading cause of major reoperation after THR [4]. Finally, as major reoperations due to POPFF are likely to have been missed with a revision-only metric, re-evaluation using major reoperation as a primary outcome is needed.

The primary aim of this study was to estimate the cumulative incidence of first-time reoperation following primary THR, which included revision for any cause or fixation of the femur for POPFF. The secondary aim was to evaluate cumulative incidence of reoperation by patient age and sex.

## Materials and methods

### Ethics statement

We reviewed prospectively collected data for all patients who had details of a primary THR submitted to the National Joint Registry (NJR). Data was accessed through the NJR research portal and analysed using R (4.2.0). Approval for the study and the planned methodology was granted by the NJR Research Committee (RSC2019/35 and RSC2019/07) prior to data access. Changes to the analysis plan were data-driven and are specified in explanation of statistical methods. Change in methodology and results were shared with the NJR research committee internal collaborator (JMW).

Hospital Episode Statistics (HES) admitted patient care data is collected on National Health Service (NHS) funded procedures performed in the NHS or the independent sector in England, but is not collected in Wales, Northern Ireland, or the Isle of Man. This study is reported as per the Reporting of studies Conducted using Observational Routinely collected health Data (RECORD) Statement (S1 RECORD Checklist).

**Fig 1:**
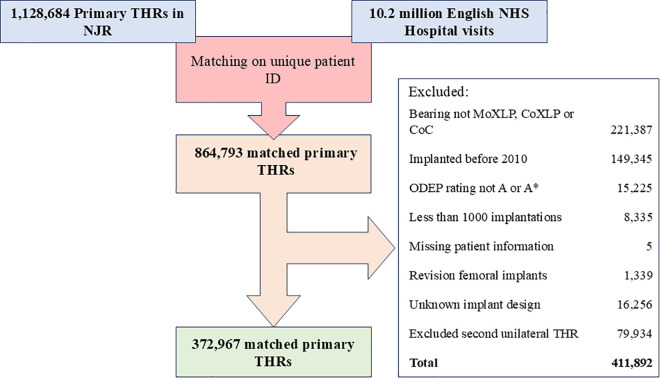
Flow chart depicting the data sources, exclusions, and final dataset. THR indicates total hip arthroplasty, ODEP indicates Orthopaedic Device Evaluation Panel and ratings were evaluated from the latest rating available in September 2024, MoXLP indicates metal on highly cross-linked polyethylene, CoXLP indicates ceramic on highly cross-linked polyethylene, and CoC indicates ceramic.

The study population was all THRs implanted in the NJR from 1 January 2010 to 31 December 2020 with data linkable to HES by unique patient identifier, and consent for their data to be used in research. Final follow-up via hospital records on 28 February 2022. Data were accessed through the NJR research portal. To improve applicability of findings to current surgical practice, cases performed using implants without the highest safety ratings were excluded. Safety ratings were taken from the Orthopaedic Device Evaluation Panel’s (ODEP’s) most recent rating given for the femoral implant in September 2024. Only implants with a rating of ODEP A* or ODEP A, indicating a low revision rate, were included. Primary THR performed with revision femoral components or unknown implants were also included (Fig 1).

A combination of International Classification of Diseases, version 10, (ICD-10) and Office of Population Censuses and Surveys Classification of Interventions and Procedures, version 4, (OPCS4) codes in the HES data were used to identify cases of revision surgery and fixation surgery for POPFF (see S1 Diagnotic Codes). Any operation identified in HES data which occurred prior to that recorded in NJR data replaced the previous outcome. Revision cases were identified using ICD-10 and OPCS4 codes already provided by the NJR for use in the annual data quality audit (See S1 Diagnostic Codes). Fixation was identified if the patient had an episode with concurrent ICD-10 codes for femoral fracture and OPCS codes relating to either fixation or revision surgery on the same side as the original THR.

### Statistical analysis

The primary outcome of this study was major reoperation (MR), defined as any revision surgery for all indications or postoperative periprosthetic femoral fracture (POPFF) treated with internal fixation (ORIF) or any operation which included both. This outcome was chosen because patients identified it as the most important, regardless of the surgery’s indication. [15] Complete case analysis was undertaken with relevant exclusions undertaken (Fig 1). Incidence was estimated using the prosthesis time incidence rate, which is the number of new events per 1,000 years that a prosthesis has been in place. Cumulative incidence of reoperation was calculated using the Kaplan–Meier method for all included THRs, censored by death or at end of follow-up on 28 February 2022. Results are given with confidence intervals (CI) of 95%. Since a large proportion of reoperation is caused by POPFF, cases were grouped by implant designs known to have strong associations with POPFF risk (Stainless steel polished taper slip [PTS], cobalt chrome PTS, composite beam [CB], cementless collared, cementless collarless) [16,17], to help surgeons understand the performance of their preferred implants according to their design. These results were stratified by age group quartiles and patient sex to highlight the reoperation outcomes associated with different implant groups. This analysis followed unexpected findings in our exploratory analysis and were data-driven. All analyses were performed using R (v 4.2.0, R, Vienna, Austria). A sensitivity analysis was conducted to assess the effect of within-patient clustering on the observations reported in this paper. A full report of findings can be found in supplemental files (S1 Sensitivity Analysis). To reduce the effect of within-patient clustering, only the first hip of bilateral sequential THR and one THR from each simultaneous bilateral THR was included.

## Results

372,967 THRs were included in the primary analysis group, with a median follow-up time of 5.39 years (range 0 to 12.1 years). 2.1% (8,043/372,967) of patients underwent reoperation over a total accumulated observation time of 2,127,464 years. 11.5% (42,880/372,967) of patients had died and 86.3% had not undergone reoperation (322,044/372,967). Of those undergoing MR, revision occurred in 1.9% (8428/372,967) and fixation of POPFF occurred in 0.2% (979/372,967) of patients. The incidence of reoperation was 3.78% (95% CI [3.70%, 3.86%]) per 1,000 prostheses years in comparison to 3.00% (95% CI [2.93%, 3.07%]) per 1,000 prostheses years when using conventional revision only outcomes.

The characteristics of the patients in our study were similar to the overall population of patients in the NJR as described in the 2023 annual report [18]. Most of our patients were women, had a mild systemic disease (American Society of Anaesthesiologists 2), and underwent THR for osteoarthritis (Table 1).

**Table 1:** Demographics of the study cohort at time of primary total hip replacement. Paediatric hip disease groups all indications for primary total hip replacement which result from childhood hip disease.

		Overall
***n***		372,967
**Age (Years) (median [IQR])**		68 [60–75]
**Sex (%)**	*Female*	217,332 (58.3)
	*Male*	155,635 (41.7)
**American Society of Anaesthesiologists grading (%)**	*Fit and healthy*	52,093 (14.0)
	*Mild disease not incapacitating*	257,835 (69.1)
	*Incapacitating systemic disease*	61,279 (16.4)
	*Life-threatening disease*	1,751 (0.5)
	*Expected to die within 24 h*	9 (0.0)
**Indication for surgery (%)**	*Acute trauma, including NOF fracture*	18,678 (5.0)
	*AVN*	9,782 (2.6)
	*Chronic trauma*	3,887 (1.0)
	*Inflammatory arthritis*	4,827 (1.3)
	*Malignancy*	377 (0.1)
	*Osteoarthritis*	324,997 (87.1)
	*Other*	2,368 (0.6)
	*Paediatric hip disease*	8,051 (2.2)
**Bilateral hip replacement (%)**	*Unilateral*	309,956 (83.1)
	*First bilateral*	54,415 (14.6)
	*Simultaneous bilateral*	8,596 (2.3)
**Stem design (%)**	*Cemented polished taper slip (Stainless steel)*	139,825 (37.5)
	*Cementless collared*	83,125 (22.3)
	*Cementless collarless*	113,196 (30.4)
	*Cemented composite beam*	2,265 (0.6)
	*Cemented polished taper slip (Cobalt chrome)*	34,556 (9.3)
**Head size (mm) (median [IQR])**		32 [32 to 36]
**Bearing (%)**	*Ceramic on ceramic*	61,270 (16.4)
	*Ceramic on highly cross-linked polyethylene*	112,547 (30.2)
	*Metal on highly cross-linked polyethylene*	199,150 (53.4)

Note: IQR indicates interquartile range, AVN indicates avascular necrosis of the femoral head, NOF indicates neck of femur fracture.

Sensitivity analysis did not demonstrate a change in the outcome of the analysis when attempting to account for within-patient clustering secondary to bilateral hip replacements (S1 Sensitivity Analysis).

**Fig 2:**
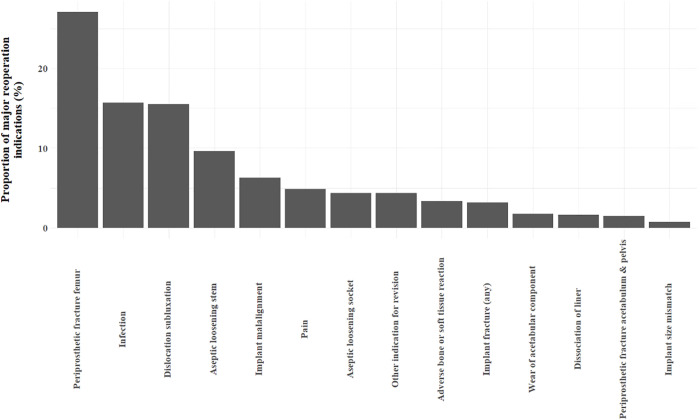
Reasons for 8,773 reoperations of total hip replacements. A breakdown of the numerical values of indications for reoperation is provided in S1 Table. ‘Other indication for revision’ is a non-descriptive option available for indications which are not otherwise categorised on data collection forms. Numerical results to accompany Fig 2 showing reasons for reoperation for all patients can be found in S1 Table.

**Fig 3:**
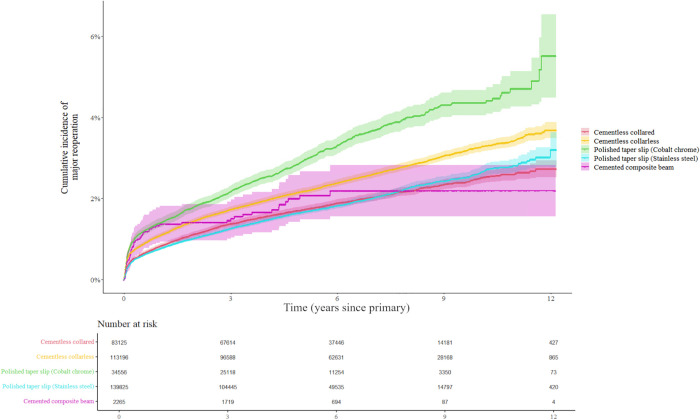
Cumulative major reoperations (unadjusted) for all patients with total hip replacement. The shaded area indicates 95% confidence intervals of the unadjusted cumulative incidence estimate.

For all patients, the most common indication for MR after hip replacement was POPFF, followed by infection and instability or dislocation (Fig 2). At 10 years, 7,968 reoperations occurred, giving a cumulative incidence of 3.1% (95% CI [3.0%, 3.1%]). Overall unadjusted 10-year cumulative incidence of MR was lowest in patients receiving a cemented CB stem, 2.1% (95% CI [1.4%, 2.8%]) or a cementless collared stem, 2.5% (95% CI [2.4%, 2.7%], Fig 3). Higher cumulative incidence of reoperation was noted in patients receiving a PTS cemented stem made of cobalt chrome 4.6% (95% CI [4.3%, 5.0%]).

**Fig 4:**
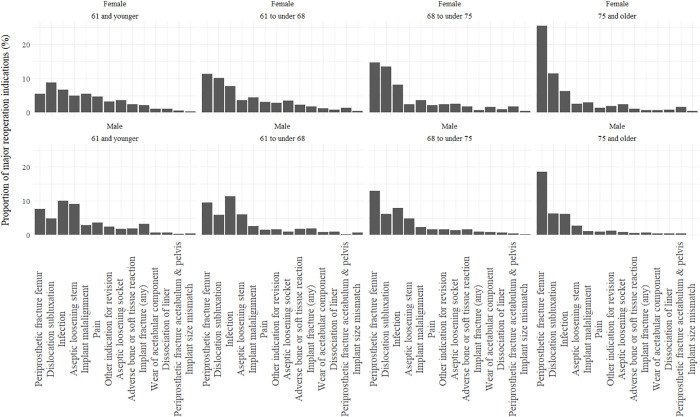
Cumulative major reoperations (unadjusted) for all patients with total hip replacement stratified by age quartiles and patient sex. The shaded area indicates 95% confidence intervals of the unadjusted cumulative incidence estimate (Composite beam removed due to very low numbers). Numbers at risk to accompany Fig 4 showing cumulative reoperation for patients stratified by age and sex can be found in S2 Table.

**Fig 5:**
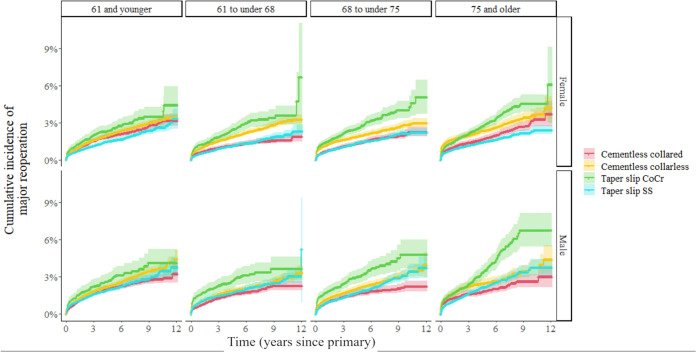
Reasons for major reoperation of total hip replacement stratified by age quartiles and patient sex. A breakdown of the numerical values of indications for reoperation is provided in S2 Table. ‘Other indication for revision’ is a non-descriptive option available for indications which are not otherwise categorised on data collection forms. Numerical results to accompany Fig 5 showing reasons for reoperation for patients stratified by age and sex can be found in S3 Table.

Patients receiving cementless femoral stems were younger and a greater proportion of these patients were men compared to the proportion of men in other groups (Table 2). After stratification for age quantile and patient sex, it can be seen for patients below the age of 68, the lowest cumulative incidence of reoperation was observed in patients receiving a cemented stainless steel PTS stem or a collared cementless stem. For older patients, there was a marked difference in cumulative incidence of reoperation between older men and women, where men aged 68 and older experienced lowest number of reoperations with a cementless collared stem and women aged 75 and older experienced lowest number of reoperations with a stainless-steel cemented PTS stem (Fig 4). POPFF was a dominant indication for revision in the oldest two quartiles of the patient cohort. In younger males, infection was more common than in younger female patients, where dislocation was more common (Fig 5). High cumulative incidence of reoperation is noted in cemented PTS stems made of cobalt chrome in all age groups and in particular older men (Fig 4).

**Table 2:** Demographics of each stem design group at time of primary total hip replacement.

	Taper slip SS	Cementless collared	Cementless collarless	Composite beam	Taper slip CoCr
***n***	139,825	83,125	113,196	2,265	34,556
**Age (Years) (median [IQR])**	71.00 [66.00, 77.00]	67.00 [60.00, 74.00]	65.00 [57.00, 72.00]	75.00 [70.00, 81.00]	72.00 [65.00, 78.00]
**Sex (%)**					
*Female*	86,754 (62.0)	48,183 (58.0)	58,896 (52.0)	1,557 (68.7)	21,942 (64.1)
*Male*	53,071 (38.0)	34,942 (42.0)	54,300 (48.0)	708 (31.3)	12,614 (36.5)
**ASA (%)**					
*Fit and healthy*	16,571 (11.9)	11,881 (14.3)	20,063 (17.7)	142 (6.3)	3,436 (9.9)
*Mild disease not incapacitating*	95,256 (68.1)	58,975 (0.9)	79,023 (69.8)	1,526 (67.4)	23,055 (66.7)
*Incapacitating systemic disease*	27,185 (19.4)	11,919 (14.3)	13,779 (12.2)	579 (25.6)	7,817 (22.6)
*Life-threatening disease*	808 (0.6)	347 (0.4)	330 (0.3)	18 (0.8)	248 (0.7)
*Expected to die within 24 h*	5 (0.0)	3 (0.0)	1 (0.0)	0 (0.0)	0 (0.0)
**Indication for surgery (%)**					
*Acute trauma, including NOF*	10,978 (7.9)	2,645 (3.2)	2,280 (2.0)	301 (13.3)	2,474 (7.2)
*AVN*	3,818 (2.7)	2,645 (3.2)	3,038 (2.7)	61 (2.7)	989 (2.9)
*Chronic trauma*	2,007 (1.2)	640 (0.6)	985 (0.7)	33 (1.2)	417 (1.2)
*Inflammatory arthritis*	2031 (1.5)	926 (1.1)	1,390 (1.2)	25 (1.1)	455 (1.3)
*Malignancy*	258 (0.2)	16 (0.0)	28 (0.0)	10 (0.4)	65 (0.2)
*Osteoarthritis*	116,878 (83.6)	75,127 (90.4)	101,834 (90.0)	1,819 (80.3)	29,339 (84.9)
*Other*	1,072 (0.8)	387 (0.5)	722 (0.6)	10 (0.4)	177 (0.5)
*Paediatric disease*	2,904 (2.1)	1,543 (1.9)	2,956 (2.6)	8 (0.4)	640 (1.9)
**Bilateral hip replacement (%)**					
*Unilateral*	119,452 (85.4)	68,178 (82.0)	90,584 (80.0)	2007 (88.6)	29,735 (86.0)
*First bilateral*	17,549 (12.6)	12,822 (15.4)	19,679 (17.4)	215 (9.5)	4,150 (12.0)
*Second bilateral*					
*Simultaneous bilateral*	2,824 (2.0)	2,125 (2.6)	2,933 (2.6)	43 (1.9)	671 (1.9)
**Head size (mm) (median [IQR])**	32.00 [28.00, 32.00]	32.00 [32.00, 36.00]	32.00 [32.00, 36.00]	32.00 [32.00,36.00]	32.00 [32.00, 36.00]
**Bearing**					
*Ceramic on ceramic*	6,920 (4.9)	20,794 (25.0)	31,362 (27.7)	44 (1.9)	2,150 (6.2)
*Ceramic on highly cross-linked polyethylene*	46,141 (33.0)	23,654 (28.5)	31,578 (27.9)	488 (21.5)	10,686 (30.9)
*Metal on highly cross-linked polyethylene*	86,764 (62.1)	38,677 (46.5)	50,256 (44.4)	1,733 (76.5)	21,720 (62.9)

Note: ASA, American Society of Anaesthesiologists; IQR indicates interquartile range, AVN indicates avascular necrosis of the femoral head, NOF indicates neck of femur fracture.

## Discussion

The inclusion of POPFF treated with fixation as an endpoint in reoperation alongside revision for all indications resulted in approximately 20% more reoperations than may have been appreciated by revision operations alone. POPFF was the most common indication for reoperation in all patients and became relatively more common as an indication in older patients. In the older quartile of patients, POPFF accounted for almost half of all re-operations. In patients 68 years old and older, stem design groups are strongly associated with the risk of major reoperation, which was also sex dependent. Patients with cemented PTS stems made with cobalt chrome had the most reoperations in comparison to other stem groups, and this observed difference was greatest in men aged 68 and older. Men aged 68 and older with cementless collared stems had better outcomes than those with cemented PTS stems made with stainless steel. Women in the older quartile with cemented PTS stems made with stainless steel fared better than those with collared cementless stems. These results contrast with current guidance, which specify age as the only criteria for implant selection regardless of patient sex.

Recent evidence has demonstrated that almost half of POPFF treated with surgery were previously uncounted by revision-only registry metrics [4], which form the basis of product and service evaluation in major joint replacement surgery. Recent changes in NJR data collection practice have sought to capture reoperations for a number of indications to include in these metrics [19], but they are likely to take many years to produce meaningful results with the necessary follow-up period to inform implant selection, given the time period most patients require a hip replacement to last. The strength of this study is that the inclusion of POPFF fixation as an outcome may increase the relevance of the results to patients who want to reduce the risk of major reoperation after THR. The key weakness of the study is that codes used to identify POPFF in the linked database have not been formally validated for sensitivity and specificity. This means that there may be over- or under-identification of cases. However, the ratio of cases treated with revision to those treated with fixation is consistent with survey data from the National Hip Fracture Database [20].

Whilst we have attempted to communicate the data in a simple, clear way using unadjusted raw cumulative incidence, differences exist between the groups which may influence the risk of reoperation between the implant groups. As a result, the findings of this study should be interpreted within the context of the patient group characteristics which are compared.

In addition, we have not yet addressed the same issue for two other key reasons for failure: infection and dislocation. The large proportion of dislocated THRs are treated with closed reduction in the emergency department and may not require hospital admission, which would not be captured in the linked data used in this study. Recent evidence suggests that the cumulative incidence of dislocation may be as high as 0.9% within 30 days, but only 11% of these patients undergo revision surgery [21]. Our patient advisory group agreed that because of the lack of bleeding, infection and morbidity associated with closed reduction, this should not be included as a major reoperation. Similarly, infected THRs treated with debridement and no implant exchange were not captured prior to June 2018. Operations involving both debridement and implant exchange would have been recorded in the NJR dataset. Reoperation for infection is often associated with large-volume blood loss [22]. Including all open operations for infection will improve the accuracy and completeness of large registry datasets. In addition, there are patients with failed THR who do not undergo operations due to excessive risks of surgery, who are also not included in this study. Inclusion of underreported reoperations will enable more accurate planning and resource allocation for future research.

In the interest of clarity for general orthopaedic readership, this study does not attempt to address the imbalance between compared groups beyond simple stratification. Modelling may be attempted to adjust for imbalances between patient groups statistically as part of further studies, but care should be taken to carefully preserve sex-implant interactions and to account for any in-person clustering for patients with bilateral joint replacement.

Inclusion of POPFF treated with fixation is likely to have increased the accuracy and validity of the findings of this study. This is in part because POPFF around cemented stems may more easily be successfully treated with fixation [6,23], leading to an imbalance of outcome reporting and subsequent performance assessment between femoral stems using revision-only datasets. The reasons behind differences in stem performance, which appear to modify POPFF risk, are maturing. Cobalt chrome as a material for cemented PTS stems has been shown to be associated with large increases in both revision risk [24] and also now reoperation in this study. The reasons for poor performance are unclear and have been the subject of recent investigation with mixed results [25–27]. Conversely, results of cemented CB stems made with cobalt chrome appear to be much better both to an endpoint of revision and reoperation for any cause [28]. Further research into optimum design to reduce the risk of POPFF is greatly needed to understand how better to protect patients in the future. Cementless stems with collars have been shown to reduce the risk of revision versus collarless cementless stems through a reduction in periprosthetic fracture risk [29,30], probably through a decrease in relative movement between the stem and the femur during an injury [31–33]. Recent results have shown very good performance of this stem class in older patients versus a mixture of cemented stem designs [34] and also against cemented PTS stems made of stainless steel [35]. This study showed that older men experienced fewer reoperations with a collared cementless stem, whereas women experienced better results with a cemented PTS stem made of stainless steel. Similar results have been observed in a large cohort study [35], but the reasons behind this remain unclear. It may be that age-related postmenopausal bone loss leads to a poorer environment for cementless stem integration than that experienced by older men. We have demonstrated a clear difference in performance of stem design groups, which are known to influence POPFF risk in older patients, where the exposure to this risk is highest. This study supports the use of implants, which reduce the risk of POPFF in older patients regardless of the presence or absence of cement. Reported differences between groups in this study may be due to unmeasured imbalances between confounding factors within the groups. Further work is required to explore these relationships and confirm treatment effects relating to implant choices in different patient groups. In addition, where patients have undergone hip replacements in both hips sequentially, these procedures may be subject to within-patient clustering, which could affect the results. Previous work has not shown a useful effect of statistical adjustments for within-patient clustering [36]. We have taken measures to account for this with inclusion of first hips in cases of sequential bilateral hip replacement to mitigate the risk of survival bias. Previous work had shown that inclusion of second hip may be preferable [37], but in our dataset, this did not make an appreciable difference to the results (S1 Sensitivity analysis).

Current guidance stipulates that surgeons should use a cemented femoral implant, without reference to design features or patient sex, in hip replacements for patients over the age of 70 years in England [9]. This has been based on registry-only data, which demonstrated better implant survival for cemented stem hip replacements in older patients [8] and has been both supported [38] and refuted [34] by findings using similar methodology. This study supports a more holistic evaluation of stem performance, which may include age, sex and implant features, so that the best outcomes can be achieved following THR, particularly in the older population. Future research should focus on prospective methods to evaluate these so that a more robust result can be obtained.

This work has demonstrated the value of comparing implants across a range of patient groups, where the risk of POPFF differs. Further work is required to explore the design features of other parts of the THR construct may affect the outcome in at-risk populations. Similar approaches should be attempted to compare the outcomes of implants designed to reduce the risk of infection or dislocation in populations where there is most risk. It is likely that a more focused patient-centred approach will demonstrate the greatest benefits, should they be evident.

More generally, surgeons should test implant selection approaches to THR to find the optimum implant choice for each patient group. Historically, some surgeons have used one implant design philosophy with excellent results [39–41], but this needs to be rigorously tested, and a more balanced data-driven approach may be beneficial.

Postoperative periprosthetic fracture of the femur dominates failure of hip replacement in older patients. The risk of major reoperation is strongly associated with the patients age, sex and implant choice. Surgeons and policy makers should re-evaluate current guidance on implant choice.

## Supporting information

S1 Diagnostic Codes Outline of methodology for identification of periprosthetic fractures using routinely collected coding data. (DOCX)

S1 Sensitivity Analysis Document detailing sensitivity analysis methodology and results. (DOCX)

S1 RECORD Checklist RECORD checklist. (PDF)

S1 Table Numerical results to accompany Fig 2 showing reasons for reoperation for all patients. (CSV)

S2 Table Patients at risk of major reoperation to accompany Fig 4 showing Cumulative major reoperations (unadjusted) for all patients with total hip replacement stratified by age quartiles and patient sex. (CSV)

S3 Table Numerical results to accompany Fig 5 showing reasons for reoperation for all patients stratified by age quartiles and patient sex. (CSV)

## Abbreviations

CB: composite beam
CI: confidence interval
HES: Hospital Episode Statistics
ICD: International Classification of Diseases
MR: major reoperation
NHS: National Health Service
NJR: National Joint Registry
OEPD: Orthopaedic Device Evaluation Panel
OPCS: Office of Population Censuses and Surveys
POPFF: postoperative periprosthetic femoral fracture
PTS: polished taper slip
RECORD: Reporting of studies Conducted using Observational Routinely collected health Data
THR: total hip replacement

## References

[1] Evans JT, Evans JP, Walker RW, Blom AW, Whitehouse MR, Sayers A. How long does a hip replacement last? A systematic review and meta-analysis of case series and national registry reports with more than 15 years of follow-up. Lancet. 2019;393(10172):647–54. doi: 10.1016/S0140-6736(18)31665-9 30782340 PMC6376618

[2] Ferguson RJ, Palmer AJ, Taylor A, Porter ML, Malchau H, Glyn-Jones S. Hip replacement. Lancet. 2018;392(10158):1662–71. doi: 10.1016/S0140-6736(18)31777-X 30496081

[3] Vanhegan IS, Malik AK, Jayakumar P, Ul Islam S, Haddad FS. A financial analysis of revision hip arthroplasty: the economic burden in relation to the national tariff. J Bone Joint Surg Br. 2012;94(5):619–23. doi: 10.1302/0301-620X.94B5.27073 22529080

[4] Lamb JN, Evans JT, Relton S, Whitehouse MR, Wilkinson JM, Pandit H. The incidence of postoperative periprosthetic femoral fracture following total hip replacement: an analysis of UK National Joint Registry and Hospital Episodes statistics data. PLoS Med. 2024;21(10):e1004462. doi: 10.1371/journal.pmed.1004462 39352892 PMC11444412

[5] Chatziagorou G, Lindahl H, Garellick G, Kärrholm J. Incidence and demographics of 1751 surgically treated periprosthetic femoral fractures around a primary hip prosthesis. Hip Int. 2019;29(3):282–8. doi: 10.1177/1120700018779558 30009622

[6] Powell-Bowns MFR, Oag E, Ng N, Pandit H, Moran M, Patton JT, et al. Vancouver B periprosthetic fractures involving the Exeter cemented stem. Bone Joint J. 2021;103-B(2):309–20. doi: 10.1302/0301-620X.103B2.BJJ-2020-0695.R1 33517729

[7] Zheng H, Gu H, Shao H, Huang Y, Yang D, Tang H, et al. Treatment and outcomes of Vancouver type B periprosthetic femoral fractures. Bone Joint J. 2020;102-B(3):293–300. doi: 10.1302/0301-620X.102B3.BJJ-2019-0935.R1 32114805

[8] Mäkelä KT, Matilainen M, Pulkkinen P, Fenstad AM, Havelin L, Engesaeter L, et al. Failure rate of cemented and uncemented total hip replacements: register study of combined Nordic database of four nations. BMJ. 2014;348:f7592. doi: 10.1136/bmj.f7592 24418635

[9] Briggs T. A national review of adult elective orthopaedic services in England: Getting it right first time. 2015.

[10] Harris WH. The problem is osteolysis. Clin Orthop Relat Res. 1995;(311):46–53. 7634590

[11] (AAOS) AAoOS. American Joint Replacement Registry (AJRR): 2022 Annual Report. Rosemont, IL: American Academy of Orthopaedic Surgeons (AAOS); 2022.

[12] Constantin H, Le M, de Steiger R, Harris IA. Operation rate is more than double the revision rate for periprosthetic femur fractures. ANZ J Surg. 2019;89(12):1647–51. doi: 10.1111/ans.15519 31674136

[13] National Joint Registry Editorial Committee and Contributors. National Joint Registry 19th Annual Report 2022. 2022. 36516281

[14] Lamb JN, West RM, Relton SD, Wilkinson JM, Pandit HG. The risk of postoperative periprosthetic femoral fracture after total hip arthroplasty depends more on stem design than cement use: an analysis of National Health Data from England. J Bone Joint Surg Am. 2025;107(5):476–87. doi: 10.2106/JBJS.24.00894 39874379

[15] Gooberman-Hill R, Burston A, Clark E, Johnson E, Nolan S, Wells V, et al. Involving patients in research: considering good practice. Musculoskeletal Care. 2013;11(4):187–90. doi: 10.1002/msc.1060 24311367 PMC3918577

[16] Lamb JN, Baetz J, Messer-Hannemann P, Adekanmbi I, van Duren BH, Redmond A, et al. A calcar collar is protective against early periprosthetic femoral fracture around cementless femoral components in primary total hip arthroplasty: a registry study with biomechanical validation. Bone Joint J. 2019;101-B(7):779–86. doi: 10.1302/0301-620X.101B7.BJJ-2018-1422.R1 31256663

[17] Lamb JN, Jain S, King SW, West RM, Pandit HG. Risk factors for revision of polished taper-slip cemented stems for periprosthetic femoral fracture after primary total hip replacement: a registry-based cohort study from the National Joint Registry for England, Wales, Northern Ireland and the Isle of Man. JBJS. 2020;102(18). 10.2106/JBJS.19.01242 32604382

[18] Ben-Shlomo Y, Blom A, Boulton C, Brittain R, Clark E, Dawson-Bowling S, et al. The National Joint Registry 20th annual report 2023. London: National Joint Registry; 2023. 38422195

[19] Registry NJ. Update of NJR minimum data set. 2024 [cited 01/08/2024]. Available from: https://www.njrcentre.org.uk/healthcare-providers/update-of-njr-minimum-data-set-forms/

[20] NHFD Facilities survey [Internet]. 2022. Available from: https://www.nhfd.co.uk/reportopen/NHFD-2023+Facilities+Survey+Results

[21] Cnudde PHJ, Nåtman J, Rolfson O, Hailer NP. The true dislocation incidence following elective total hip replacement in Sweden: how does it relate to the revision rate? J Clin Med. 2024;13(2):598. doi: 10.3390/jcm13020598 38276104 PMC10816596

[22] Sharqzad AS, Cavalheiro C, Zahar A, Lausmann C, Gehrke T, Kendoff D, et al. Blood loss and allogeneic transfusion for surgical treatment of periprosthetic joint infection: a comparison of one- vs. two-stage exchange total hip arthroplasty. Int Orthop. 2019;43(9):2025–30. doi: 10.1007/s00264-018-4137-y 30187096

[23] Smitham PJ, Carbone TA, Bolam SM, Kim YS, Callary SA, Costi K, et al. Vancouver B2 peri-prosthetic fractures in cemented femoral implants can be treated with open reduction and internal fixation alone without revision. J Arthroplasty. 2019;34(7):1430–4. doi: 10.1016/j.arth.2019.03.003 30956048

[24] Lamb JN, King SW, Cage ES, van Duren BH, West RM, Pandit HG. Factors affecting risk of periprosthetic fracture revision of cemented polished taper stems: a design linked registry analysis from the national joint registry of England, Wales and the Isle of Man. In: British Orthopaedic Association Conference, 10/09/2019. Liverpool: 2019.

[25] Jain S, Lamb JN, Drake R, Entwistle I, Baren JP, Thompson Z, et al. Risk factors for periprosthetic femoral fracture risk around a cemented polished taper-slip stem using an osteoporotic composite bone model. Proc Inst Mech Eng H. 2024;238(3):324–31. doi: 10.1177/09544119231225172 38235693

[26] Yagura T, Oe K, Kobayasi F, Sogawa S, Nakamura T, Iida H, et al. Experimental periprosthetic fractures with collarless polished tapered cemented stems. Int Orthop. 2024;48(5):1171–8. doi: 10.1007/s00264-024-06136-1 38443715

[27] Kaneuji A, Chen M, Takahashi E, Takano N, Fukui M, Soma D, et al. Collarless polished tapered stems of identical shape provide differing outcomes for stainless steel and cobalt chrome: a biomechanical study. J Funct Biomater. 2023;14(5):262. doi: 10.3390/jfb14050262 37233372 PMC10219186

[28] Mukka S, Mellner C, Knutsson B, Sayed-Noor A, Sköldenberg O. Substantially higher prevalence of postoperative peri-prosthetic fractures in octogenarians with hip fractures operated with a cemented, polished tapered stem rather than an anatomic stem. Acta Orthop. 2016;87(3):257–61. doi: 10.3109/17453674.2016.1162898 27045318 PMC4900095

[29] Melbye SM, Haug SCD, Fenstad AM, Furnes O, Gjertsen J-E, Hallan G. How does implant survivorship vary with different Corail femoral stem variants? Results of 51,212 cases with up to 30 years of follow-up from the Norwegian Arthroplasty Register. Clin Orthop Relat Res. 2021;479(10):2169–80. doi: 10.1097/CORR.0000000000001940 34427568 PMC8445552

[30] Rele S, O’Bryan E, Holder C, Lewis PL, Di Bella C. Collared cementless femoral components reduce the revision rates in primary total hip arthroplasty using the direct anterior approach: an Australian Orthopaedic Association National Joint Replacement Registry Study. J Arthroplasty. 2024;39(9S2):S340–6.e2. doi: 10.1016/j.arth.2024.05.009 38735543

[31] Johnson AJ, Desai S, Zhang C, Koh K, Zhang L-Q, Costales T, et al. A calcar collar is protective against early torsional/spiral periprosthetic femoral fracture: a paired cadaveric biomechanical analysis. J Bone Joint Surg Am. 2020;102(16):1427–33. doi: 10.2106/JBJS.19.01125 32816417

[32] Lamb JN, Baetz J, Messer-Hannemann P, Adekanmbi I, van Duren BH, Redmond A, et al. A calcar collar is protective against early periprosthetic femoral fracture around cementless femoral components in primary total hip arthroplasty: a registry study with biomechanical validation. Bone Joint J. 2019;101-B(7):779–86. doi: 10.1302/0301-620X.101B7.BJJ-2018-1422.R1 31256663

[33] Demey G, Fary C, Lustig S, Neyret P, si Selmi TA. Does a collar improve the immediate stability of uncemented femoral hip stems in total hip arthroplasty? A bilateral comparative cadaver study. J Arthroplasty. 2011;26(8):1549–55. doi: 10.1016/j.arth.2011.03.030 21570801

[34] Orce Rodríguez A, Smith PN, Johnson P, O’Sullivan M, Holder C, Shimmin A. Registry-based study of survivorship of cemented femoral components versus collared cementless femoral components in total hip arthroplasty in older patients with osteoarthritis. Bone Joint J. 2024;106-B(3 Supple A):121–9. doi: 10.1302/0301-620X.106B3.BJJ-2023-0771.R1 38423086

[35] Lynch Wong M, Robinson M, Bryce L, Cassidy R, Lamb JN, Diamond O, et al. Reoperation risk of periprosthetic fracture after primary total hip arthroplasty using a collared cementless or a taper-slip cemented stem. Bone Joint J. 2024;106-B(2):144–50. doi: 10.1302/0301-620X.106B2.BJJ-2023-0739.R1 38425304

[36] Lie SA, Engesaeter LB, Havelin LI, Gjessing HK, Vollset SE. Dependency issues in survival analyses of 55,782 primary hip replacements from 47,355 patients. Stat Med. 2004;23(20):3227–40. doi: 10.1002/sim.1905 15449328

[37] Bülow E, Nemes S, Rolfson O. Are the first or the second hips of staged bilateral THAs more similar to unilateral procedures? A study from the Swedish Hip Arthroplasty Register. Clin Orthop Relat Res. 2020;478(6):1262–70. doi: 10.1097/CORR.0000000000001210 32168059 PMC7319399

[38] Bloemheuvel EM, Van Steenbergen LN, Swierstra BA. Comparable mortality but higher revision rate after uncemented compared with cemented total hip arthroplasties in patients 80 years and older: report of 43,053 cases of the Dutch Arthroplasty Register. Acta Orthop. 2022;93:151–7. doi: 10.2340/17453674.2021.886 34984473 PMC8815327

[39] Purbach B, Kay PR, Siney PD, Fleming PA, Wroblewski BM. The C-stem in clinical practice: fifteen-year follow-up of a triple tapered polished cemented stem. J Arthroplasty. 2013;28(8):1367–71. doi: 10.1016/j.arth.2012.10.030 23528555

[40] Westerman RW, Whitehouse SL, Hubble MJW, Timperley AJ, Howell JR, Wilson MJ. The Exeter V40 cemented femoral component at a minimum 10-year follow-up: the first 540 cases. Bone Joint J. 2018;100-B(8):1002–9. doi: 10.1302/0301-620X.100B8.BJJ-2017-1535.R1 30062940

[41] Vidalain J-P. Twenty-year results of the cementless Corail stem. Int Orthop. 2011;35(2):189–94. doi: 10.1007/s00264-010-1117-2 20814676 PMC3032112

