# Supplementary material for: The association between implant design, age, sex and the rate of major reoperation in patients undergoing primary total hip replacement: A retrospective study of UK National Joint Registry and Hospital Episodes Statistics data
Source: PLoS Med. 2025 Nov 26;22(11):e1004538. doi: 10.1371/journal.pmed.1004538 (PMC12680321; doi:10.1371/journal.pmed.1004538)
Supplement: S1 Diagnostic Codes — (DOCX) [file pmed.1004538.s001.docx]

Codes used in identifying fractures

**Diagnosis of POPFF**

ICD code + OPCS 4 (fixation or revision) + side match THR primary + date before known outcome date.

**ICD code:**

M96.6 or M97.0 or S72 or M97.1 or M97.9 or M84.45 or M80.05 or M80.85 or M80.95 or M84.35 or M84.55 or M84.65 or M84.75 or M90.75

**OPCS fixation codes:**

Any of (W19 or W201 or W202 or W203 or W204 or W208 or W209 or W24 or O17 or W22 or W23 or W24 or W25 or W26 or W65 or W66 or W67) with femur code (Z76 or Z843)

Or any of (O171 or W191 or W241)

**OPCS revision codes:**

[*https://www.njrcentre.org.uk/wp-content/uploads/OPCS-Procedure-codes-relevant-to-NJRv8-njrcentre-Healthcare-providers-Entering-data-Manual-and-training.pdf*](https://www.njrcentre.org.uk/wp-content/uploads/OPCS-Procedure-codes-relevant-to-NJRv8-njrcentre-Healthcare-providers-Entering-data-Manual-and-training.pdf)

| NJR code | opcs1 | opcs2 | Text |
| --- | --- | --- | --- |
| H2.1 | W370 | W372 | Conversion from previous cemented total prosthetic replacement of hip joint \|Conversion to total prosthetic replacement of hip joint using cement |
| H2.2 | W370 | W382 | Conversion from previous cemented total prosthetic replacement of hip joint \|Conversion to total prosthetic replacement of hip joint not using cement |
| H2.3 | W370 | W392 | Conversion from previous cemented total prosthetic replacement of hip joint \| Conversion to total prosthetic replacement of hip joint NEC |
| H2.4 | W370 | W932 | Conversion from previous cemented total prosthetic replacement of hip joint \|Conversion to hybrid prosthetic replacement of hip joint using cemented acetabular component |
| H2.5 | W370 | W942 | Conversion from previous cemented total prosthetic replacement of hip joint \|Conversion to hybrid prosthetic replacement of hip joint using cemented femoral component |
| H2.6 | W370 | W952 | Conversion from previous cemented total prosthetic replacement of hip joint \|Conversion to hybrid prosthetic replacement of hip joint using cement NEC |
| H2.7 | W373 |  | Revision of total prosthetic replacement of hip joint using cement |
| H2.8 | W374 |  | Revision of one component of total prosthetic replacement of hip joint using cement |
| H2.9 | W380 | W372 | Conversion from previous uncemented total prosthetic replacement of hip joint \|Conversion to total prosthetic replacement of hip joint using cement |
| H2.10 | W380 | W382 | Conversion from previous uncemented total prosthetic replacement of hip joint \|Conversion to total prosthetic replacement of hip joint not using cement |
| H2.11 | W380 | W392 | Conversion from previous uncemented total prosthetic replacement of hip joint \|Conversion to total prosthetic replacement of hip joint NEC |
| H2.12 | W380 | W932 | Conversion from previous uncemented total prosthetic replacement of hip joint\| Conversion to hybrid prosthetic replacement of hip joint using cemented acetabular component |
| H2.13 | W380 | W942 | Conversion from previous uncemented total prosthetic replacement of hip joint \| Conversion to hybrid prosthetic replacement of hip joint using cemented femoral component |
| H2.14 | W380 | W952 | Conversion from previous uncemented total prosthetic replacement of hip joint \| Conversion to hybrid prosthetic replacement of hip joint using cement NEC |
| H2.15 | W383 |  | Revision of total prosthetic replacement of hip joint not using cement |
| H2.16 | W384 |  | Revision of one component of total prosthetic replacement of hip joint not using cement |
| H2.17 | W390 | W372 | Conversion from previous total prosthetic replacement of hip joint NEC \|Conversion to total prosthetic replacement of hip joint using cement |
| H2.18 | W390 | W382 | Conversion from previous total prosthetic replacement of hip joint NEC \|Conversion to total prosthetic replacement of hip joint not using cement |
| H2.19 | W390 | W392 | Conversion from previous total prosthetic replacement of hip joint NEC \|Conversion to total prosthetic replacement of hip joint NEC |
| H2.20 | W390 | W932 | Conversion from previous total prosthetic replacement of hip joint NEC \| Conversion to hybrid prosthetic replacement of hip joint using cemented acetabular component |
| H2.21 | W390 | W942 | Conversion from previous total prosthetic replacement of hip joint NEC \| Conversion to hybrid prosthetic replacement of hip joint using cemented femoral component |
| H2.22 | W390 | W952 | Conversion from previous total prosthetic replacement of hip joint NEC \| Conversion to hybrid prosthetic replacement of hip joint using cement NEC |
| H2.23 | W393 |  | Revision of total prosthetic replacement of hip joint NEC |
| H2.24 | W394 | Y032 | Attention to total prosthetic replacement of hip joint NEC \| Renewal of prosthesis in organ NOC |
| H2.25 | W394 | Y036 | Attention to total prosthetic replacement of hip joint NEC \| Adjustment to prosthesis in organ NOC |
| H2.26 | W395 |  | Revision of one component of total prosthetic replacement of hip joint NEC |
| H2.27 | W580 | W372 | Conversion from previous resurfacing arthroplasty of joint \| Conversion to total prosthetic replacement of hip joint using cement |
| H2.28 | W580 | W382 | Conversion from previous resurfacing arthroplasty of joint \| Conversion to total prosthetic replacement of hip joint not using cement |
| H2.29 | W580 | W392 | Conversion from previous resurfacing arthroplasty of joint \| Conversion to total prosthetic replacement of hip joint NEC |
| H2.30 | W580 | W932 | Conversion from previous resurfacing arthroplasty of joint \| Conversion to hybrid prosthetic replacement of hip joint using cemented acetabular component |
| H2.31 | W580 | W942 | Conversion from previous resurfacing arthroplasty of joint \| Conversion to hybrid prosthetic replacement of hip joint using cemented femoral component |
| H2.32 | W580 | W952 | Conversion from previous resurfacing arthroplasty of joint \| Conversion to hybrid prosthetic replacement of hip joint using cement NEC |
| H2.33 | W582 | Z843 | Revision of resurfacing arthroplasty of joint \| Hip Joint |
| H2.34 | W582 | Z902 | Revision of resurfacing arthroplasty of joint \| Hip NEC |
| H2.35 | W930 | W372 | Conversion from previous hybrid prosthetic replacement of hip joint using cemented acetabular component \| Conversion to total prosthetic replacement of hip joint using cement |
| H2.36 | W930 | W382 | Conversion from previous hybrid prosthetic replacement of hip joint using cemented acetabular component \| Conversion to total prosthetic replacement of hip joint not using cement |
| H2.37 | W930 | W392 | Conversion from previous hybrid prosthetic replacement of hip joint using cemented acetabular component \| Conversion to total prosthetic replacement of hip joint NEC |
| H2.38 | W930 | W932 | Conversion from previous hybrid prosthetic replacement of hip joint using cemented acetabular component \| Conversion to hybrid prosthetic replacement of hip joint using cemented acetabular component |
| H2.39 | W930 | W942 | Conversion from previous hybrid prosthetic replacement of hip joint using cemented acetabular component \| Conversion to hybrid prosthetic replacement of hip joint using cemented femoral component |
| H2.40 | W930 | W952 | Conversion from previous hybrid prosthetic replacement of hip joint using cemented acetabular component \| Conversion to hybrid prosthetic replacement of hip joint using cement NEC |
| H2.41 | W933 |  | Revision of hybrid prosthetic replacement of hip joint using cemented acetabular component |
| H2.42 | W940 | W372 | Conversion from previous hybrid prosthetic replacement of hip joint using cemented femoral component \| Conversion to total prosthetic replacement of hip joint using cement |
| H2.43 | W940 | W382 | Conversion from previous hybrid prosthetic replacement of hip joint using cemented femoral component \| Conversion to total prosthetic replacement of hip joint not using cement |
| H2.44 | W940 | W392 | Conversion from previous hybrid prosthetic replacement of hip joint using cemented femoral component \| Conversion to total prosthetic replacement of hip joint NEC |
| H2.45 | W940 | W932 | Conversion from previous hybrid prosthetic replacement of hip joint using cemented femoral component \| Conversion to hybrid prosthetic replacement of hip joint using cemented acetabular component |
| H2.46 | W940 | W942 | Conversion from previous hybrid prosthetic replacement of hip joint using cemented femoral component \| Conversion to hybrid prosthetic replacement of hip joint using cemented femoral component |
| H2.47 | W940 | W952 | Conversion from previous hybrid prosthetic replacement of hip joint using cemented femoral component \| Conversion to hybrid prosthetic replacement of hip joint using cement NEC |
| H2.48 | W943 |  | Revision of hybrid prosthetic replacement of hip joint using cemented femoral component |
| H2.49 | W950 | W372 | Conversion from previous hybrid prosthetic replacement of hip joint using cement NEC \|Conversion to total prosthetic replacement of hip joint using cement |
| H2.50 | W950 | W382 | Conversion from previous hybrid prosthetic replacement of hip joint using cement NEC \|Conversion to total prosthetic replacement of hip joint not using cement |
| H2.51 | W950 | W392 | Conversion from previous hybrid prosthetic replacement of hip joint using cement NEC \|Conversion to total prosthetic replacement of hip joint NEC |
| H2.52 | W950 | W932 | Conversion from previous hybrid prosthetic replacement of hip joint using cement NEC \|Conversion to hybrid prosthetic replacement of hip joint using cemented acetabular component |
| H2.53 | W950 | W942 | Conversion from previous hybrid prosthetic replacement of hip joint using cement NEC \|Conversion to hybrid prosthetic replacement of hip joint using cemented femoral component |
| H2.54 | W950 | W952 | Conversion from previous hybrid prosthetic replacement of hip joint using cement NEC \|Conversion to hybrid prosthetic replacement of hip joint using cement NEC |
| H2.55 | W953 |  | Revision of hybrid prosthetic replacement of hip joint using cement NEC |
| H2.56 | W954 | Y032 | Attention to hybrid prosthetic replacement of hip joint using cement NEC \| Renewal of prosthesis in organ NOC |
| H2.57 | W370 | W372 | Conversion from previous cemented total prosthetic replacement of hip joint \|Conversion to total prosthetic replacement of hip joint using cement |

**Side codes:**

Z941*, Z942, Z943
