# Supplementary material for: The association between implant design, age, sex and the rate of major reoperation in patients undergoing primary total hip replacement: A retrospective study of UK National Joint Registry and Hospital Episodes Statistics data
Source: PLoS Med. 2025 Nov 26;22(11):e1004538. doi: 10.1371/journal.pmed.1004538 (PMC12680321; doi:10.1371/journal.pmed.1004538)
Supplement: S1 Sensitivity Analysis — (DOCX) [file pmed.1004538.s002.docx]

# Sensitivity analysis

Registry data may be subject to clustering because of repeated records on the same patients (bilateral hip replacements), which can affect the standard error of the sample and give inaccurate results when standard error is used for the basis of statistical comparison. This is not the case in this analysis. Hips can exist as first and only hip, or the second of two hip replacements, or one of two hip replacements done simultaneously (in the same operation). Since many hips performed prior to the registry records may exist, it is not possible to ascertain whether the first or second hip is observed when a patient has only one hip in the register. Only that for patients with unilateral hip replacement, their hip is the last recorded hip. To attempt to reduce the hip duplicity problem we have only included the last recorded hip replacement. We have excluded the first of sequential bilateral hips and a random selection of hips from simultaneous bilateral operations (one from each pair). The results from this sub-group are described below.

# Results

Following exclusion of first unilateral hips and a random sample of 50% of simultaneous bilateral hips, to reduce the effect of order of hip surgery in patients with hips replaced on both sides, 389,890 THRs were included in the primary analysis group, with a median follow up time of 5.05 years (range 0.0 to 12.2 years). 2.1% (8,283/ 389,890) of patients underwent reoperation over a total accumulated observation time of 2,093,203 years. 11.7% (45,117/ 389,890) of patients had died and 86.2% had not undergone reoperation (332,192/ 389,890). Of those undergoing MR, revision occurred in 1.8% (7304 /389,890) and fixation of POPFF occurred 0.3% (979 /389,890) of patients. The incidence of reoperation was 4.0% (95% confidence interval [CI 3.9%,4.0%]) per 1000 prostheses years in comparison to 3.2% (95% CI [3.1%,3.2%]) per 1000 prostheses years when using conventional revision only outcomes.

The characteristics of the patients in our study were similar to the overall population of patients in the NJR as described in the 2023 annual report[18]. Most of our patients were women, had a mild systemic disease (ASA 2), and underwent THR for osteoarthritis (Table A in S1 Sensitivity analysis).

**Table A in S1 Sensitivity analysis.** Demographics of the study cohort at time of primary total hip replacement. Paediatric hip disease groups all indications for primary total hip replacement which result from childhood hip disease. *Note: IQR indicates interquartile range, AVN indicates avascular necrosis of the femoral head, NOF indicates neck of femur fracture.*

|  |  | Overall |
| --- | --- | --- |
| n |  | 389890 |
| Age (Years) (median [IQR]) |  | 69.00 [61.00, 76.00] |
| Sex (%) | *Female* | 228,030 (58.5) |
|  | *Male* | 161,860 (41.5) |
| American Society of Anaesthesiologists grading (%) | *Fit and healthy* | 51,329 (13.2) |
|  | *Mild disease not incapacitating* | 270,036 (69.3) |
|  | *Incapacitating systemic disease* | 66,634 (17.1) |
|  | *Life threatening disease* | 1,880 (0.5) |
|  | *Expected to die within 24hrs* | 11 (0.0) |
| Indication for surgery (%) | *Acute trauma including NOF fracture* | 18,900 (4.8) |
|  | *AVN* | 9,661 (2.5) |
|  | *Chronic trauma* | 3,926 (1.0) |
|  | *Inflammatory arthritis* | 4,932 (1.3) |
|  | *Malignancy* | 380 (0.1) |
|  | *Osteoarthritis* | 341,707 (87.6) |
|  | *Other* | 2,338 (0.6) |
|  | *Paediatric hip disease* | 8,046 (2.1) |
| Bilateral hip replacement (%) | *Unilateral* | 309,956 (79.5) |
|  | *Second bilateral* | 71,338 (18.3) |
|  | *Simultaneous bilateral* | 8,596 (2.2) |
| Stem design (%) | *Cemented polished taper slip (Stainless steel)* | 148,730 (38.1) |
|  | *Cementless collared* | 86,740 (22.2) |
|  | *Cementless collarless* | 114,631 (29.4) |
|  | *Cemented composite beam* | 2,450 (0.6) |
|  | *Cemented polished taper slip (Cobalt chrome)* | 37,339 (9.6) |
| Head size (mm) (median [IQR]) |  | 32 [32 to 36] |
| Bearing (%) | *Ceramic on ceramic* | 59,941 (15.4) |
|  | *Ceramic on highly cross-linked polyethylene* | 118,475 (30.4) |
|  | *Metal on highly cross-linked polyethylene* | 211,474 (54.2) |

**Table B in S1 Sensitivity analysis.** Demographics of each stem design group at time of primary total hip replacement. *Note: IQR indicates interquartile range, AVN indicates avascular necrosis of the femoral head, NOF indicates neck of femur fracture.*

|  | **Taper slip SS** | **Cementless collared** | **Cementless collarless** | **Composite beam** | **Taper slip CoCr** |
| --- | --- | --- | --- | --- | --- |
| **n** | 148,730 | 86740 | 114631 | 2450 | 37339 |
| **Age (Years) (median [IQR])** | 71.00 [64.00, 78.00] | 68.00 [60.00, 75.00] | 65.00 [57.00, 72.00] | 76.00 [70.00, 81.00] | 72.00 [65.00, 79.00] |
| **Sex (%)** |  |  |  |  |  |
| *Female* | 92611 (62.3) | 50330 (58.0) | 59605 (52.0) | 1691 (69.0) | 23793 (63.7) |
| *Male* | 56119 (37.7) | 36410 (42.0) | 55026 (48.0) | 759 (31.0) | 13546 (36.3) |
| **ASA (%)** |  |  |  |  |  |
| *Fit and healthy* | 16653 (11.2) | 11702 (13.5) | 19308 (16.8) | 148 (6.0) | 3518 (9.4) |
| *Mild disease not incapacitating* | 101501 (68.2) | 61836 (71.3) | 80209 (70.0) | 1636 (66.8) | 24854 (66.6) |
| *Incapacitating systemic disease* | 29709 (20.0) | 12822 (14.8) | 14771 (12.9) | 645 (26.3) | 8687 (23.3) |
| *Life threatening disease* | 862 (0.6) | 377 (0.4) | 341 (0.3) | 21 (0.9) | 279 (0.7) |
| *Expected to die within 24hrs* | 5 (0.0) | 3 (0.0) | 2 (0.0) | 0 (0.0) | 1 (0.0) |
| **Indication for surgery (%)** |  |  |  |  |  |
| *Acute trauma including NOF* | 11140 (7.5) | 2664 (3.1) | 2264 (2.0) | 314 (12.8) | 2518 (6.7) |
| *AVN* | 3803 (2.6) | 1825 (2.1) | 2970 (2.6) | 56 (2.3) | 1007 (2.7) |
| *Chronic trauma* | 1929 (1.3) | 604 (0.7) | 939 (0.8) | 33 (1.3) | 421 (1.1) |
| *Inflammatory arthritis* | 2095 (1.4) | 934 (1.1) | 1403 (1.2) | 24 (1.0) | 476 (1.3) |
| *Malignancy* | 256 (0.2) | 19 (0.0) | 32 (0.0) | 10 (0.4) | 63 (0.2) |
| *Osteoarthritis* | 125458 (84.4) | 78773 (90.8) | 103434 (90.2) | 1992 (81.3) | 32050 (85.8) |
| *Other* | 1079 (0.7) | 372 (0.4) | 694 (0.6) | 13 (0.5) | 180 (0.5) |
| *Paediatric disease* | 2970 (2.0) | 1549 (1.8) | 2895 (2.5) | 8 (0.3) | 624 (1.7) |
| **Bilateral hip replacement (%)** |  |  |  |  |  |
| *Unilateral* | 119452 (80.3) | 68178 (78.6) | 90584 (79.0) | 2007 (81.9) | 29735 (79.6) |
| *Second bilateral* | 26438 (17.8) | 16456 (19.0) | 21141 (18.4) | 398 (16.2) | 6905 (18.5) |
| *Simultaneous bilateral* | 2840 (1.9) | 2106 (2.4) | 2906 (2.5) | 45 (1.8) | 699 (1.9) |
| **Head size (mm) (median [IQR])** | 32.00 [28.00, 32.00] | 32.00 [32.00, 36.00] | 32.00 [32.00, 36.00] | 32.00 [32.00,36.00] | 32.00 [32.00, 36.00] |
| **Bearing** |  |  |  |  |  |
| *Ceramic on ceramic* | 6723 (4.5) | 20630 (23.8) | 30375 (26.5) | 47 (1.9) | 2166 (5.8) |
| *Ceramic on highly cross-linked polyethylene* | 48816 (32.8) | 25032 (28.9) | 32425 (28.3) | 541 (22.1) | 11661 (31.2) |
| *Metal on highly cross-linked polyethylene* | 93191 (62.7) | 41078 (47.4) | 51831 (45.2) | 1862 (76.0) | 23512 (63.0) |

Figure A in S1 Sensitivity analysis shows the that the most common indication for reoperation was POPFF followed by dislocation and infection.


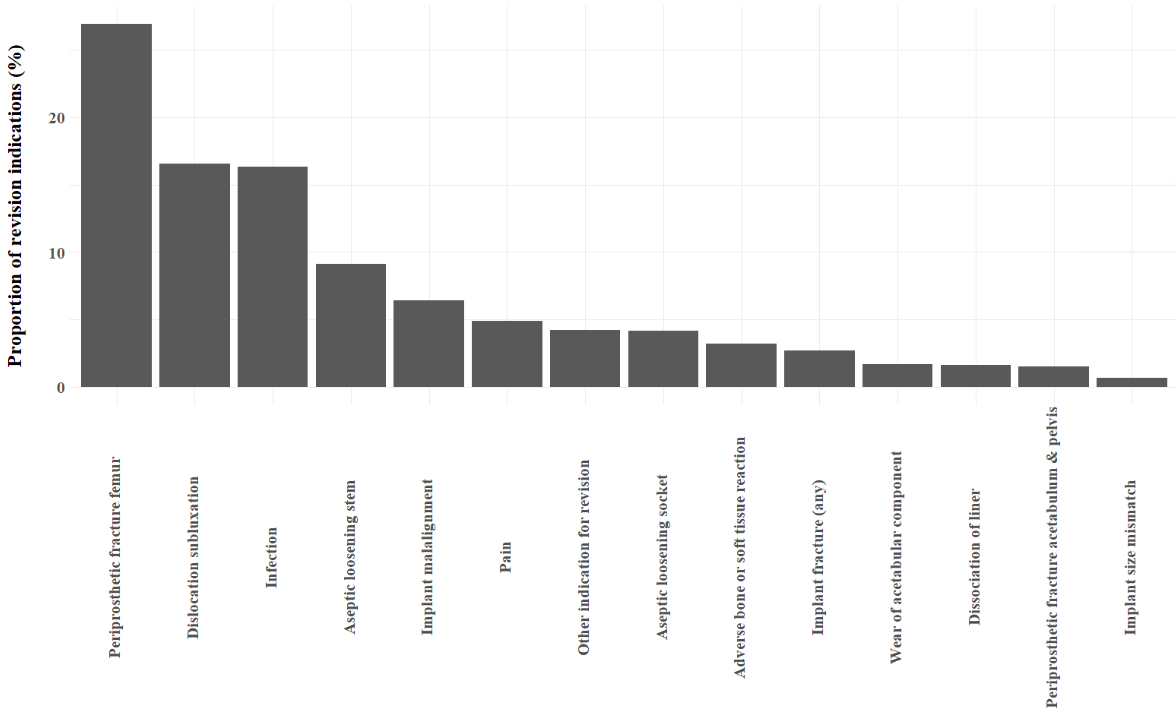


**Figure A in S1 Sensitivity analysis.** Reasons for reoperation of total hip replacements.


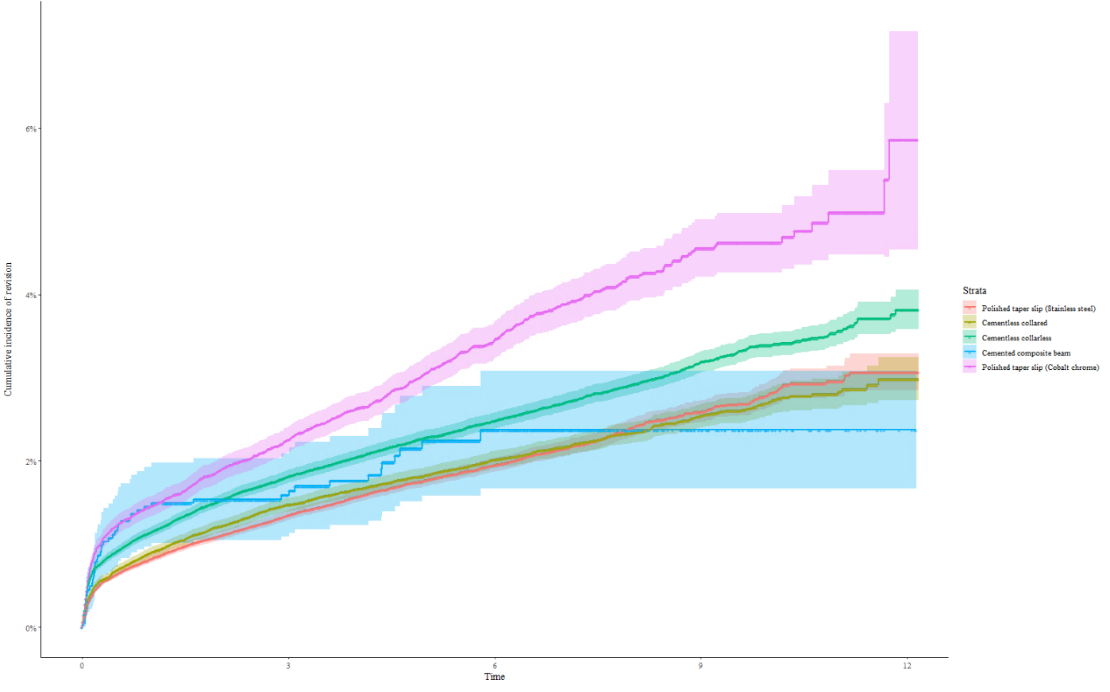


**Figure B in S1 Sensitivity analysis.** Cumulative reoperations (unadjusted) for all patients with total hip replacement. The shaded area indicates 95% confidence intervals of the unadjusted cumulative incidence estimate.

For all patients the most common indication for MR after hip replacement was POPFF followed by infection and instability or dislocation (Figure A in S1 Sensitivity analysis). At ten years 9,325 reoperation occurred giving a cumulative incidence of 3.0% (95% CI [2.9%,3.0%]). Overall unadjusted cumulative incidence was lowest in patients receiving a cemented composite beam stem or a cementless collared stem (Figure B in S1 Sensitivity analysis). Higher cumulative incidence of reoperation was noted in patients receiving a PTS cemented stem made of cobalt chrome.

Patients receiving cementless femoral stems were younger and a greater proportion of these patients were men (Table B in S1 Sensitivity analysis). After stratification for age quantile and patient sex, it can be seen for patients below the age of 68 the lowest cumulative incidence of reoperation was observed in patients receiving a cemented stainless steel PTS stem or a collared cementless stem. For older patients there was a marked difference in cumulative incidence of reoperation between older men and women, where older men experienced least risk of reoperation with a cementless collared stem and older women experienced least risk of reoperation with a stainless-steel cemented PTS stem (Figure C in S1 Sensitivity analysis). POPFF was a dominant indication for revision in the older half of the patient cohort. In younger males, infection was more common than for younger female patients, where dislocation was more common (Figure D in S1 Sensitivity analysis). High cumulative incidence of reoperation is noted in cemented PTS stems made of cobalt chrome in all age groups and in particular older men.


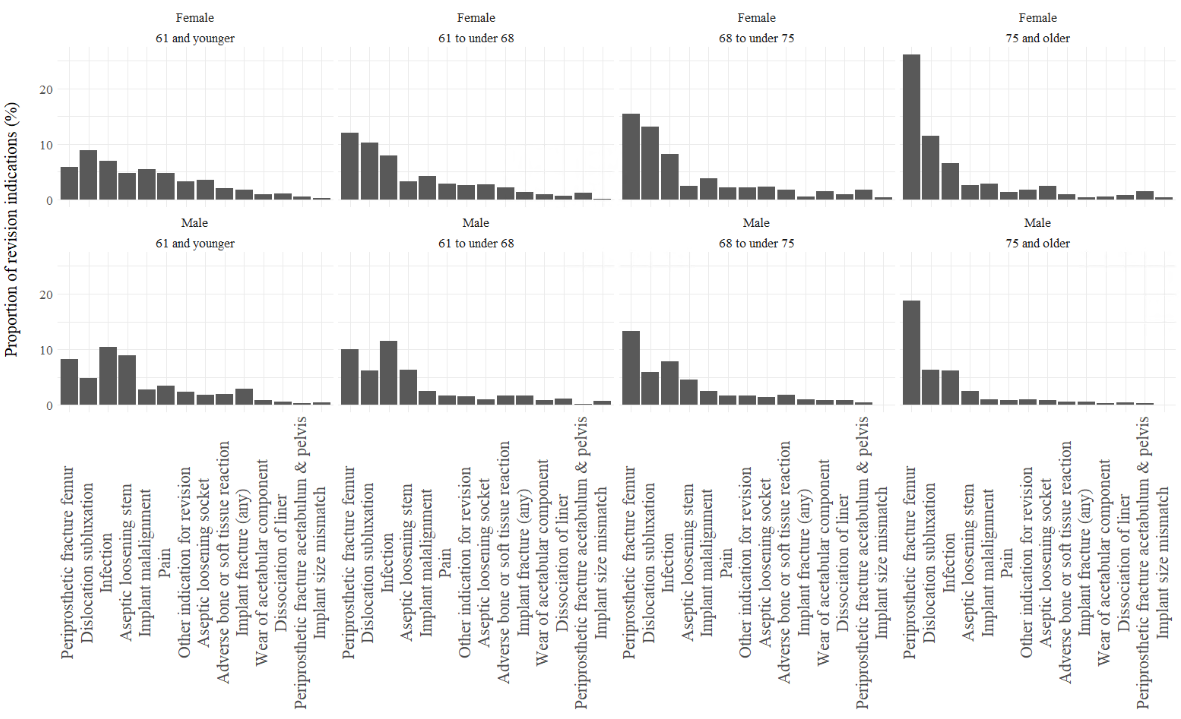
**Figure C in S1 Sensitivity analysis.** Reasons for major reoperation of total hip replacement stratified by age quartiles and patient sex.


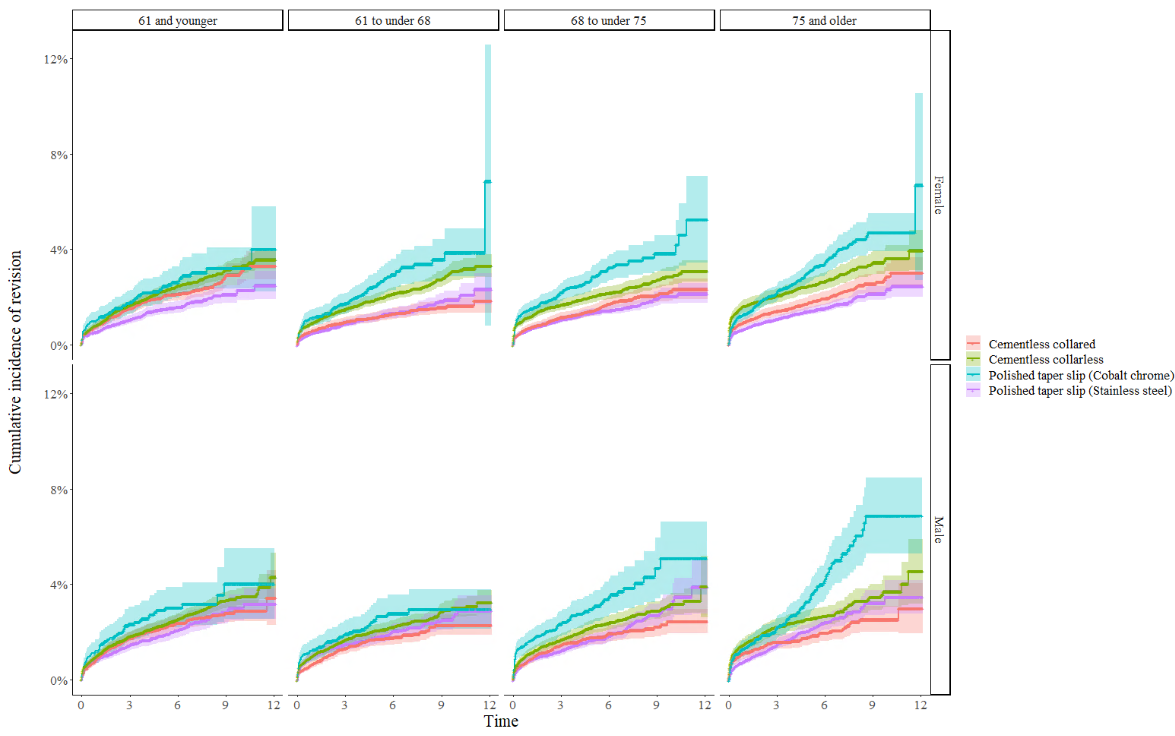


**Figure D in S1 Sensitivity analysis.** Cumulative major reoperations (unadjusted) for all patients with total hip replacement stratified by age quartiles and patient sex. *The shaded area indicates 95% confidence intervals of the unadjusted cumulative incidence estimate.*
